# Molecular Mechanisms of Anti-Melanogenic Gedunin Derived from Neem Tree (*Azadirachta indica*) Using B16F10 Mouse Melanoma Cells and Early-Stage Zebrafish

**DOI:** 10.3390/plants10020330

**Published:** 2021-02-09

**Authors:** Hwang-Ju Jeon, Kyeongnam Kim, Chaeeun Kim, Myoung-Jin Kim, Tae-Oh Kim, Sung-Eun Lee

**Affiliations:** 1 Department of Applied Biosciences, Kyungpook National University, Daegu 41566, Korea; jeonhj@knu.ac.kr (H.-J.J.); kn1188@knu.ac.kr (K.K.); myoung_jin@knu.ac.kr (M.-J.K.); 2 Department of Integrative Biology Kyungpook National University, Daegu 41566, Korea; dkrkek01@knu.ac.kr; 3 College of Civil and Environmental Engineering, Kumoh National Institute of Technology, Gumi 39253, Korea; tokim@kumoh.ac.kr; 4 Department of Energy Engineering Convergence, Kumoh National Institute of Technology, Gumi 39177, Korea; 5 Department of Environmental Engineering, Kumoh National Institute of Technology, Gumi 39177, Korea

**Keywords:** MITF, microphthalmia-associated transcription factor, TYR, tyrosinase, DQ, dopaquinone, TRP-1, tyrosinase-related protein 1, TRP-2, tyrosinase-related protein 2, MC1R, melanocortin 1 receptor, α-MSH, α-melanocyte stimulating hormone, ACTH, adrenocorticotropic hormone, ASP, agouti signaling protein, cAMP, cyclic adenosine monophosphate, CREB, cAMP response element

## Abstract

Melanogenesis represents a series of processes that produce melanin, a protective skin pigment (against ultraviolet rays), and determines human skin color. Chemicals reducing melanin production have always been in demand in the cosmetic market because of skincare interests, such as whitening. The main mechanism for inhibiting melanin production is the inhibition of tyrosinase (TYR), a key enzyme for melanogenesis. Here, we evaluated gedunin (Ged), a representative limonoid, for its anti-melanogenesis action. Melanin production in vitro was stimulated by alpha-melanocyte stimulating hormone (α-MSH) in B16F10 mouse melanoma cells. Ged reduced α-MSH-stimulated melanin production, inhibiting TYR activity and protein amount. We confirmed this result in vivo in a zebrafish model for melanogenesis. There was no sign of toxicity and malformation of zebrafish embryos during development in all treated concentrations. Ged reduced the number of produced zebrafish embryo pigment dots and melanin contents of embryos. The highly active concentration of Ged (100 µM) was much lower than the positive control, kojic acid (8 mM). Hence, Ged could be a fascinating candidate for anti-melanogenesis reagents.

## 1. Introduction

Melanin is synthesized in the melanosome, lysosome-like organelles of the melanocyte. In the melanosome, melanin is produced in two pigments, eumelanin and pheomelanin, representing brown-black and red-yellow, respectively [1]. The synthesis of these melanin pigments starts from the precursor, L-tyrosine, with oxidation reaction of L-tyrosine into a compound called dopaquinone (DQ), and L-DOPA [2]. In this oxidation step, tyrosinase (TYR) acts as a rate limiting enzyme of the overall melanin biosynthesis pathway [2,3]. TRP-1 and TRP-2, closely related to melanogenesis proteins, catalyze the eumelanin biosynthesis pathway and stabilize and induce TYR activity [1]. The process producing melanin pigments (called melanogenesis) occurs in the melanosome of melanocytes, a site for melanin synthesis and storage [4]. In melanogenesis signaling pathway, melanocortin 1 receptor (MC1R) is a member of G-protein-coupled receptors. MCIR is a starting point for melanogenesis signaling and is activated by MC1R agonists like α-melanocyte stimulating hormone (α-MSH), adrenocorticotropic hormone (ACTH), and agouti signaling protein (ASP) [1,2]. The activation of α-MSH-MC1R signaling pathway increases cyclic adenosine monophosphate (cAMP) concentration and phosphorylates the cAMP response element (CREB). Phosphorylated CREB induces the protein level of microphthalmia-associated transcription factor (MITF), a transcription factor regulating melanin-synthesis-related genes, TYR, tyrosinase-related protein-1 (TRP-1), and tyrosinase-related protein-2 (TRP-2) by binding the M-box of these genes [1,5,6]. Among the five melanocortin receptors, MC1R has the most abundant melanocytes. It primarily regulates eumelanin (black-brown pigment) production by activating MC1R when this receptor’s agonists, such as a-MSH and ACTH, are switched to this receptor [2,4,7].

Although there are differences between mammals and fishes, zebrafish have an extra copy of MC5R and MC3R, which the pufferfish does not possess [7], and this signaling pathway still exists in zebrafish and works exactly as it does in mammal melanocytes [7]. Moreover, the number of studies on the inhibition of melanin pigment formation has increased due to its research advantages, such as rapid development in early-stage embryos and ease of observation [8,9,10,11]. In these phenomena, many previous studies have found that the inhibition of this signaling pathway reduces melanin production and found several inhibitors, including bisabolangelone, 4-hydroxy-3-methoxycinnamaldehyde, and diphenylmethylenehydrazine carbothiamide [12,13,14].

Gedunin (Ged), a well-known heat-shock protein 90 inhibitor, is a limonoid, found in Meliaceae plant’s genera. This limonoid is abundant primarily in the epicarp of young Indian neem tree (*Azadirachta indica*) fruits and has diverse biological activities, including anticancer, antimalarial, anti-inflammation, antidiabetic, antiallergic, insecticidal, herbicidal, and antifungal [15,16,17,18]. There is no report about the mechanism of the anti-melanogenic effect of Ged.

As a medicinal drug or cosmetic agent candidate to treat melanin-related diseases or skin color changes, Ged might have advantages because of its safe, naturally occurring plant properties [19]. The anti-melanogenic effect of Ged were evaluated in vitro and in vivo using B16F10 mouse melanoma cells and zebrafish embryos, respectively, to find a proper whiting agent candidate, one of the largest parts of the commercial cosmetic market. Simultaneously, through zebrafish, an acute toxicity test was also performed to show how Ged possibly affects animals.

## 2. Material and Methods

### 2.1. Chemicals and Reagents

Ged was purchased from Microsource Discovery Systems, Inc. (Gayloardville, CT, USA). Melanogenesis stimulator, α-MSH, anti-melanogenic compound, and kojic acid were purchased from Sigma-Aldrich (St. Louis, MO, USA). All other reagents were higher than molecular biology grade.

### 2.2. Cell Culture

The mouse melanocarcinoma cell line, B16F10, was obtained from the American Type Culture Collection (ATCC, Manassas, VA, USA) and cultured with 10% fetal bovine serum (*v*/*v*), and penicillin supplied Dulbecco-modified Eagle’s media (GE Healthcare, Chicago, IL, USA) at a humidified atmosphere with 5% CO2. Cells were cultured until confluency reached 80% and divided three times a week with a dividing ratio of 1:8. Cells were observed every 12 h, and morphological changes were checked. The passage number of used cells was kept small.

### 2.3. Cell Viability Assay

To evaluate the proliferative effect of Ged on B16F10 mouse melanoma cell line, MTS assay was performed according to previously reported protocol [20] using CellTiter 96 Aqueous One Solution Cell Proliferation Assay kit (Promega, Madison, WI, USA). Briefly, 1 × 103 cells were seeded in each well of the 96-well plate and incubated for 24 h for recovery. After incubation, Ged was treated with various concentrations: 0, 3.12, 6.25, 12.5 25, 50, and 75 μM. After 48 h, 20 μL of MTS assay solution was added to each well containing 100-μL media with Ged and without Ged. After an additional incubation for 4 h, absorbance was measured at 490 nm using Multiskan GO spectrophotometer (Thermo Scientific, Waltham, MA, USA). The absorbance data were normalized with the control and represented as a percentage inhibition ratio.

### 2.4. Determination of Melanin Contents

Extracellular and intracellular melanin contents were determined following a modified method previously reported [21]. α-MSH (200 nM) pretreated melanoma cells were incubated for 72 h with and without kojic acid (200 μM) and Ged (25 and 50 μM). After incubation, phenol-red free culture media was collected, and cultured cells were harvested with RIPA lysis buffer. Harvested cells were centrifuged at 4 °C, 13,000 rpm for 15 min, and pellets were dissolved in 1 M NaOH, 10% DMSO solution at 95 °C for 2 h. The absorbance of collected media and dissolved melanin was measured at 470 nm using a spectrophotometer. All samples were normalized with their protein concentration determined following the BCA method.

### 2.5. Intracellular Tyrosinase Activity Assay

Cellular tyrosinase activity was determined by measuring the oxidation rate of L-dihydroxyphenylalanine (L-DOPA) as previously reported, with slight modifications [22]. B16F10 mouse melanoma cells were pretreated with 200 nM of α-MSH for 1 h followed by treatment with 200 μM kojic acid, with and without Ged (25 and 50 μM), and incubated for 72 h. After incubation, cells were harvested with lysis buffer containing proteinase inhibitor, and the sample was centrifuged at 13,000× *g*, 4 °C for 15 min. The supernatant was transferred to a new tube. Each sample (20 μL) and 80 μL of 40 mM L-DOPA were mixed in a 96-well plate and incubated at 37 °C for 2 h. Absorbance at 475 nm by oxidated L-DOPA was determined using a microplate reader. The absorbance value was normalized with the protein concentration of each sample.

### 2.6. RNA Isolation and qRT-PCR

Total RNA isolation and qRT-PCR were done according to a method previously described by [18]. Total RNA was briefly extracted from each sample using Trizol reagent (Ambion, Austin, TX, USA). Extracted total RNA concentration was determined by absorbance at 260 nm, and the quality of total RNA was checked with an absorbance ratio of 260:280 and 260:230 nm using Multiskan GO microplate reader (Thermo Scientific). Total RNA (5 μg) was synthesized into cDNA with Maxima first strand cDNA synthesis kit (Thermo Scientific) and synthesized cDNA. mRNA expression levels of Mitf, Tyr, Trp-1, and Trp-2 were determined using Luna Universal qPCR Master Mix (New England Biolabs, Ipswich, MA, USA) and QuantStudio 3 Real-Time PCR instrument (Applied Biosystems, Foster City, CA, USA) according to instructor’s protocol. The expression level of genes was normalized with glyceraldehyde 3-phosphate dehydrogenase (GAPDH).

### 2.7. Immunoblot Analysis

Immunoblot analysis was performed according to the methods previously described [23]. Proteins of B16f10 cells were collected using CETi lysis buffer (Translab, Daejeon, Korea), and the concentration was determined with Pierece BCA protein assay kit (Thermo Scientific). An equal amount of proteins (30 µg) of each sample were loaded onto a sodium dodecyl sulfate-polyacrylamide gel and electrophoresed. After separation, proteins were transferred to a nitrocellulose membrane, and the membrane was blocked in 10% nonfat skim milk solution for 2 h. Blocked membranes were incubated with primary antibody overnight at 4 °C, followed by another incubation with secondary antibody at 26 °C for 2 h. Primary and secondary antibodies were diluted in a ratio of 1:1000 and 1:3000, respectively. Lastly, blotted membranes were documented using ChemiDoc (Bio-Rad, Hercules, CA, USA) with ECL reagent (SuperSignal, Thermo Scientific). Primary and secondary antibodies were purchased from Santa Cruz Biotechnology (Dallas, TX, USA).

### 2.8. Zebrafish Embryo Test

Wild-type zebrafish was thankfully obtained from Professor Tae-Lin Huh, the School of Life Science and Biotechnology, Kyungpook National University, Daegu, Republic of Korea. The strain was kept for more than eight generations in a laboratory condition of 26 °C ± 1 °C, and a day-to-night ratio of 8:16. General fish care and breeding conditions, as previously described, were followed [24]. Ten groups of zebrafish were mated to obtain embryos with a mating ratio (male:female, 1:2), and among obtained embryos, healthy embryos had >80% fertilization rate and were selected and used for the experiment. Twelve healthy embryos in each treated group were located in each well of a 96-well plate with and without Ged (25, 50, 75, and 100 μM) and kojic acid (8 mM) in E3 media at shield stage. At 24 h post-fertilization (hpf), embryos were dechorionated and abnormality was checked every 24 h until 72 hpf. The embryos’ phenotype was photographed in a lateral and dorsal view to compare differences between groups using BX53 upright microscope with DP80 CCD (Olympus Life Science Solutions, Waltham, MA, USA). After picturing, embryos were homogenized with CETi lysis buffer (TransLab), and determination of melanin concentration was performed, which was equal to B16F10 cell melanin content determination.

### 2.9. Statistical Analysis

All statistical analyses presented in this study were performed using GraphPad Prism v.8.0 (GraphPad Software, San Diego, CA, USA). One-way ANOVA with Tukey’s test was used for multiple comparisons. All data are represented as the mean ± the standard deviations. Significant differences were considered statistically significant at *p* < 0.05.

## 3. Results

### 3.1. Cytotoxic Effect of Piperlongumine in B16F10 Cells

**Figure 1:**
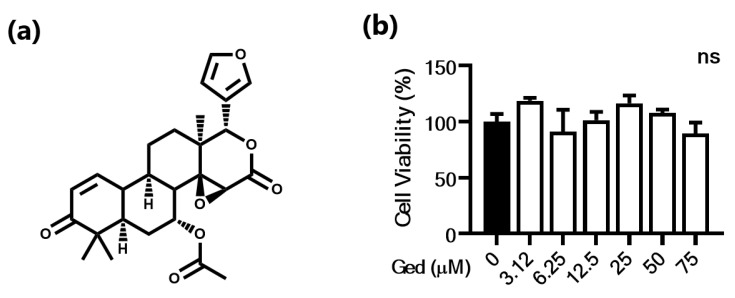
Cytotoxicity of gedunin (Ged) in B16F10 cells. (**a**) Chemical structure of Ged. (**b**) Viability of B16F10 cells with the treatment of 3.12, 6.25, 12.5, 25, 50, and 70 µM of Ged and without it. Data are presented as means ± standard deviation (SD) of triplicate experiments.

Before investigating the anti-melanogenic effect of Ged, we performed a viability test in the B16F10 cell line to set the dose range in this study. In the concentration range from 3.12 to 50 μM, cells did not show any inhibitory effect on growth, and their relative proliferation was from 91.0% to 118.7% (Figure 1). In contrast, although there was no statistical significance, the 75 μM-treated group had their growth slightly inhibited (89.3%; Figure 1). Therefore, the highest concentration was set to 50 μM in the remaining cell-line experiments.

### 3.2. Gedunin Inhibits Melanin Production and Intracellular Tyrosinase Activity in B16F10 Mouse Melanoma Cells

**Figure 2:**
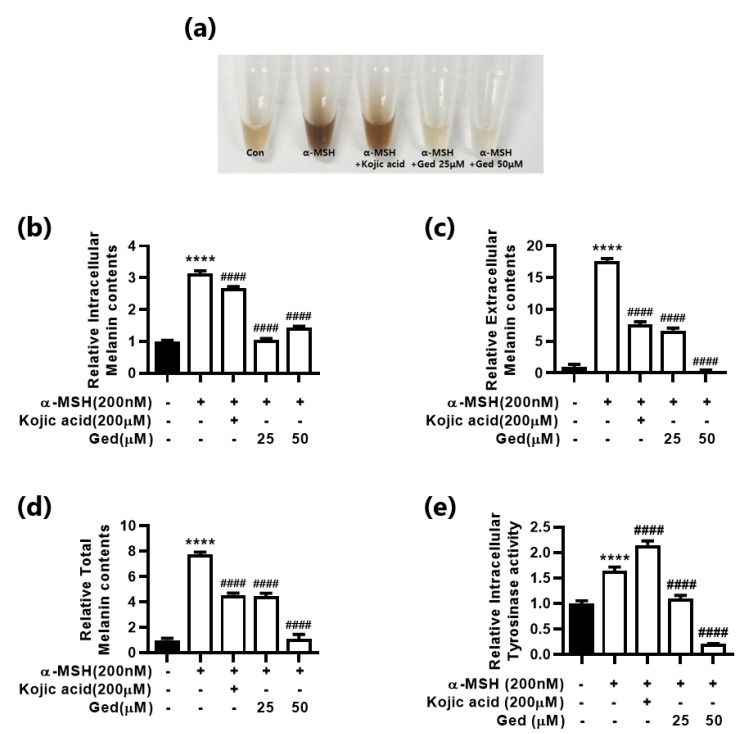
Anti-melanogenic effect of gedunin (Ged) on alpha-melanocyte stimulating hormone (α-MSH)-stimulated melanin production in B16F10 cells. (**a**) The color of the phenol-red free culture media after 48 h treatment of α-MSH, kojic acid, and Ged as stimuli, positive control substance, and testing substance, respectively. (**b**) Intracellular and (**c**) extracellular melanin contents in B16F10 cells and two values were pooled and (**d**) designated as total melanin content. (**e**) Tyrosinase activity in the extracted protein sample. Data are presented as means ± standard deviation (SD) of triplicate experiments. **** *p* < 0.0001 compared with control and #### *p* < 0.0001 compared with α-MSH-stimulated group.

We evaluated the anti-melanogenic effect of Ged in B16F10 mouse melanoma cells. The MC1R agonist, α-MSH, effectively induced intracellular and extracellular melanin production (Figure 2a–c). The color of the collected media was dark with α-MSH (Figure 2a). The color was faded with the addition of Ged, and the effect was dose-dependent (Figure 2a). In the results of total melanin contents, α-MSH increased melanin production in melanocytes approximately seven-fold higher than the control group and stimulated melanin decrease by adding kojic acid, a well-known positive control in melanogenesis (Figure 2d). Ged also shortened stimuli-inducing melanin production in melanocytes at all concentrations tested in this study (Figure 2d). Moreover, in the tyrosinase activity test, Ged showed a strong inhibitory effect on the rate-limiting enzyme, tyrosinase, in melanogenesis (Figure 2e). The relative activity of tyrosinase in a 50 μM Ged treated group decreased by 20% and 12.24% compared with the control and a-MSH-stimulated group, respectively.

### 3.3. Gedunin Reduces α-MSH Induced Melanogenesis-Related Gene Expression

**Figure 3:**
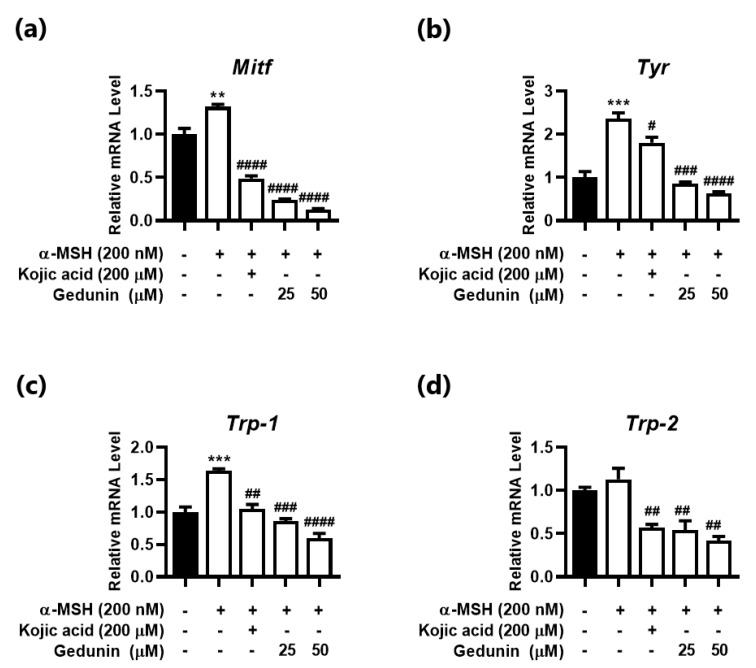
Effect of gedunin on melanogenesis-related genes. mRNA level of genes determined by quantitative PCR. (**a**) Microphthalmia-associated transcription factor (Mitf), 6 h after treatment. (**b**) Tyrosinase (Tyr), (**c**) tyrosinase-related protein-1 (Trp-1), and (**d**) tyrosinase-related protein-2 (Tyr-2), 48 h after treatment. Data are presented as means ± standard deviation (SD) of triplicates. ** *p* < 0.01 and *** *p* < 0.001, compared with control, and # *p* < 0.05, ## *p* < 0.01, ### *p* < 0.001, and #### *p* < 0.0001, compared with α-MSH stimulated group.

α-MSH (200nM) induced mRNA level of Mitf; a melanogenesis transcription factor; and target genes Tyr, Trp-1, and Trp-2 (Figure 3). Treatment of Ged reduced mRNA level of all the genes in a dose-dependent manner at the concentration of 25 and 50 μM (Figure 3). Compared with the positive control, kojic acid (200 μM), Ged was more effective reducing mRNA level of melanogenesis related genes in B16F10 cells. Furthermore, the level of Mitf and Tyr decreased by 0.10- and 0.26-fold lower than α-MSH induced cells, respectively, at a concentration of 50 μM (Figure 3a,b). Down-regulated mRNA levels of these genes lowered the level of *Tyr* and reduced the amount of TYR production of melanin less than control group.

### 3.4. Gedunin Reduced TYR and TYR-1 Amount

**Figure 4:**
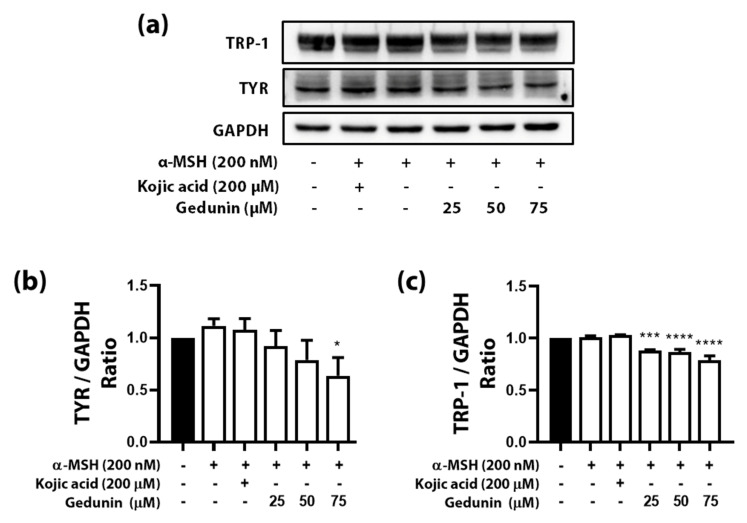
Western blotting of alpha-melanocyte stimulating hormone (α-MSH) induced B16F10 cells with gedunin treatment. (**a**) Band images of Western blotting of proteins such as tyrosinase-related protein-1 (TRP-1), tyrosinase (TYR), and glyceraldehyde 3-phosphate dehydrogenase (GAPDH). (**b**) Relative protein level of TYR; (**c**) relative protein level of TRP-1. Graphs were drawn based on extracted data using ImageJ software (ImageJ, U.S. National Institutes of Health, Bethesda, MD, US). Data are presented as means ± standard deviation (SD) of triplicate experiments. * *p* < 0.05, *** *p* < 0.001 and **** *p* < 0.0001 compared with control.

TYR plays a crucial role in melanogenesis, and the protein level of this enzyme affects melanogenesis directly. We tried confirming the alteration of the amount of protein, TYR, caused by reduced mRNA level through immunoblot analysis. TYR was noticeably decreased compared with control, and α-MSH was treated dose-dependently (Figure 4a). Moreover, TRP-1, an essential transcription factor of TYR, was slightly reduced (Figure 4a). The results were more apparent when each protein’s band was normalized by the house-keeping protein, GAPDH (Figure 4b,c).

### 3.5. Toxicity of Gedunin against Zebrafish in Early-Stage Development

**Figure 5:**
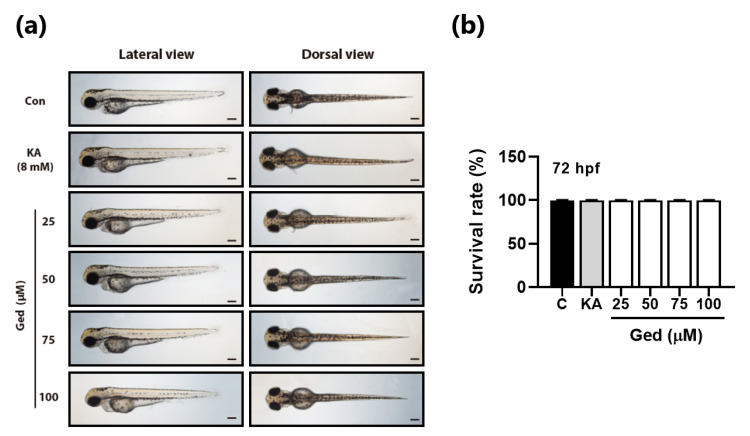
Toxicity of gedunin (Ged) on zebrafish early-stage development. (**a**) Optical images of lateral view and dorsal view of the zebrafish after 72 h of treatment of kojic acid (8 mM) and various Ged concentrations. (**b**) The survival rate of zebrafish embryos after 72 h of treatment of kojic acid and Ged.

Toxicological evaluation of Ged on zebrafish early-stage development was performed before evaluating an anti-melanogenic test of Ged in vivo. Morphological abnormality of zebrafish embryos was not observed in any treated concentration of Ged (Figure 5a). In tested concentrations of Ged, 25, 50, 75, and 100 μM, the Ged-treated groups’ survival rate had no significant differences at 72 h of the chemical-treated period (Figure 5b).

### 3.6. Gedunin Reduced Melanin Contents in Zebrafish Embryos

**Figure 6:**
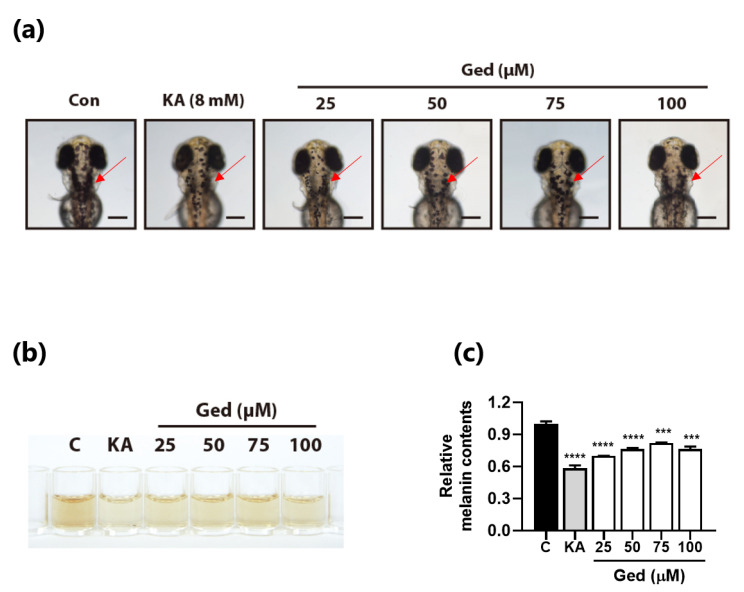
Effect of gedunin on melanin production in vivo. (**a**) Optical images of overhead view zebrafish embryos. Pigment dots were indicated with arrows. (**b**) Color of whole embryo lysate with 1 M NaOH. (**c**) Determined zebrafish embryos melanin contents by absorbance method of melanin at 490 nm. Data are presented as means ± standard deviation (SD) of triplicates. *** *p* < 0.001 and **** *p* < 0.0001.

Because Ged has no developmental toxicity in zebrafish embryos, we examined the anti-melanogenic effect at series of concentrations of Ged, 25, 50, 75, and 100 μM. In the optical observation, we checked the dots of pigment zebrafish; the overhead side view of zebrafish embryos is shown in Figure 5a. Kojic acid has an inhibition effect on pigment production of zebrafish embryos. Ged-treated zebrafish embryos also had fewer pigment dots on the head (Figure 6a). In addition, total melanin contents extracted from the whole body of zebrafish embryos decreased after Ged exposure for 72 h in both kojic acid, a positive control, and Ged-treated groups (Figure 6b,c).

## 4. Discussion

In this study, inhibition properties of Ged against melanin production were evaluated. Based on our findings, the inhibition ability of Ged was approximately 11.98-fold higher than that of kojic acid (Figure 2), and the concentration of Ged (50 μM) was lower than that of kojic acid (200 μM), the positive control. It was matched with weakened intracellular tyrosinase activity, an enzyme that converts L-Dopa to DQ, the primary precursor of eumelanin and pheomelanin. Decreased Tyr activity entails the slowing down of the entire melanin production processes and the shortage of both end products, eumelanin and pheomelanin [1,2,3]. The transcript and TYR level were regulated by a series of transcription factors: Mitf, Trp-1, and Trp-2 [1,2]. A reduced amount of protein and mRNA level means activated MC1R via α-MSH–MC1R–PKA–CREB signaling axis by α-MSH was downregulated with the treatment of Ged at a concentration of 25 and 50 μM, dose-dependently in both quantitative PCR and Western blotting. As described previously, decreasing the amount of MITF leads to shortage of Tyr, Trp-1, and Trp-2 via less binding to the promoter region of these genes [25,26,27]. Reducing these transcription factors leads TYR to a lower level and inhibits melanin pigment production in B16F10 cells. In addition, current results demonstrate the TYR inhibitory effect of Ged, another critical point of melanin production in the melanocyte. Ged also showed an inhibition effect on pigment production during zebrafish early-stage development in the in vitro and in vivo models. Our results showed that treatment with Ged for 72 h markedly reduced zebrafish embryo pigmentation. Total melanin content and mRNA levels of related genes were reduced with the presence of Ged, and the tendency of these results strongly supported the in vitro anti-melanogenesis property of Ged.

Moreover, inhibition ability of Ged was much stronger than kojic acid, one of the most commonly known compounds as a whitening reagent in cosmetic industry [6,28]. The concentration at which Ged acted was relatively lower than kojic acid. In addition, previous report showed that another common whitening reagent, arbutin, had a similar anti-melanogenic effect as kojic acid [2]. Therefore, Ged should be considered as a whitening reagent in replacement of the currently used arbutin or kojic acid, and as an anti-melanoma drug in regard to its promising properties.

Anti-melanogenic properties of Ged were suggested before [29,30], but these studies were not focused on the specific mechanisms of the effect, and they simply observed alteration of cytotoxic effects and melanin production amount in vitro, whereas anti-proliferative activity and inhibitory effects on HSP 90 expression have been highlighted [31,32,33]. The possibility of Ged as an anti-melanogenetic agent has been reported; however, these studies were not focused on the specific mechanisms of the effect, and they simply observed alteration of cytotoxic effects and melanin production amount in vitro. We tried determining the cellular level mechanism by confirming the mRNA level and protein amount of melanin production-related genes. Together with previous reports, this study showed a new perspective of Ged as a component of cosmetic ingredients for two benefits, outstanding anticancer and anti-melanogenesis, which might be applied to both UV-induced melanoma and accumulation of pigment, especially, at the same time.

## 5. Conclusions

In conclusion, all these results showed a novel compound, Ged, may be used as a depigmentation reagent for melanin biosynthesis, which shortened downstream proteins TYR, TRP-1, and TRP-2 by a reduced amount compared the master regulation protein, MITF, in both in vitro and in vivo systems of zebrafish model.

## Data Availability

Data sharing not applicable.
